# Genome-Wide Association of Genetic Variants With Refraction, Axial Length, and Corneal Curvature: A Longitudinal Study of Chinese Schoolchildren

**DOI:** 10.3389/fgene.2020.00276

**Published:** 2020-03-25

**Authors:** Yaoyao Lin, Yu Ding, Dandan Jiang, Chunchun Li, Xiaoqiong Huang, Linjie Liu, Haishao Xiao, Balamurali Vasudevan, Yanyan Chen

**Affiliations:** ^1^School of Optometry and Ophthalmology, Wenzhou Medical University, Wenzhou, China; ^2^The Eye Hospital, Wenzhou Medical University, Wenzhou, China; ^3^College of Optometry, Midwestern University, Glendale, AZ, United States

**Keywords:** genetic variants, schoolchildren myopia, association study, spherical equivalent refraction, axial length, corneal curvature

## Abstract

**Background:**

Myopia is a common eye disorder that is approaching epidemic proportions worldwide. A genome-wide association study identified *AREG* (rs12511037), *GABRR1* (rs13215566), and *PDE10A* (rs12206610) as being associated with refractive error in Asian populations. The present study investigated the associations between these three genetic variants and the occurrence and development of myopia, spherical equivalent refraction (SER), axial length (AL), and corneal curvature (CC) in a cohort of southeastern Chinese schoolchildren.

**Methods:**

We examined and followed 550 children in grade 1 enrolled in the Wenzhou Epidemiology of Refractive Error (WERE) project. During the 4-year follow-up, non-cycloplegic refraction was evaluated twice each year, and the AL and CC were measured once every year. Age, sex, and the amounts of time spent on near work and outdoors were documented with a questionnaire. Sanger DNA sequencing was used to genotype single nucleotide polymorphisms (SNPs). SNPtest software was used to identify potential genetic variants associated with myopia, SER, AL, and CC. Ten thousand permutations were used to correct for multiple testing.

**Results:**

In total, 469 children, including 249 (53.1%) boys and 220 (46.9%) girls, were included in analyses. The mean age of all the children was 6.33 ± 0.48 years. After adjusting for age, sex, time spent on near work and time spent outdoors, neither the genotypes nor the allele frequencies of the three SNPs were significantly associated with myopic shift, incident myopia or the change in SER. After adjusting for age, sex, near-work time and outdoor time with 10,000 permutations, the genotype *AREG* (rs12511037) was associated with an increase in AL (*P*′-values for the dominant, recessive, additive and general models were 0.0032, 0.0275, 0.0045, and 0.0099, respectively); the genotype *PDE10A* (rs12206610) was associated with a change in CC in the additive (*P*′ = 0.0096), dominant (*P*′ = 0.0096), and heterozygous models (*P*′ = 0.0096).

**Conclusion:**

These findings preliminarily indicate that *AREG* SNP rs12511037 and *PDE10A* SNP rs12206610 are etiologically relevant for ocular traits, providing a basis for further exploration of the development of myopia and its molecular mechanism. However, elucidating the role of *AREG* and *PDE10A* in the pathogenesis of myopia requires further animal model and human genetic epidemiology studies. This trial is registered as ChiCTR1900020584 at www.Chictr.org.cn.

## Introduction

Myopia (nearsightedness) is a common eye disorder that is reaching epidemic proportions worldwide (Williams et al., 2015; Wang et al., 2018; Tideman et al., 2019). The global prevalence of myopia is increasing and expected to increase from one in four persons in 2000 to nearly half of the global population by 2050 (Holden et al., 2016). It is generally characterized by axial elongation of the eye, accompanied by structural changes in the choroid and retina. High myopia increases the risk of complications including myopic macular degeneration, glaucoma, and cataract, all of which lead to visual impairment and even blindness (Verhoeven et al., 2015). Therefore, the prevention and treatment of myopia are important for public health.

Myopia is caused by a complex interaction between nature and nurture (Miraldi, 2017). Although numerous epidemiological studies have implicated environmental factors, most notably outdoor exposure and near work (He et al., 2015; Huang et al., 2015; Guo et al., 2017; Lin et al., 2017), as being associated with the development of myopia, it is well established that genetic factors also play an important role. At present, 161 candidate genetic loci influencing refractive error have been identified (Tedja et al., 2018). The few studies performed in the Asian population have determined the potential genetic susceptibility loci for refractive error, including *GJD2* (Cheng et al., 2013; Miyake et al., 2015), *AKAP13* (Cornes et al., 2012), *LAMA2* (Cheng et al., 2013), and *WNT7B* (Miyake et al., 2015), etc. Fan et al. (2016) reported that three novel loci, *AREG* (rs12511037), *GABRR1* (rs13215566), and *PDE10A* (rs12206610), were associated with refractive error only in the Asian population. *AREG* is involved in extracellular matrix remodeling (Duncan and Collison, 2003). *GABRR1* and *PDE10A* play a role in retinal neurotransmission and circadian rhythm, respectively (Wolloscheck et al., 2011; Fan et al., 2016).

Although *AREG* (rs12511037), *GABRR1* (rs13215566), and *PDE10A* (rs12206610) are genetic susceptibility loci for refractive error in the Asian adult population, it is unknown whether these three genes are associated with refractive error in schoolchildren. Furthermore, to the best of our knowledge, whether variants of these genes are related to refraction development, axial length (AL) and corneal curvature (CC) has not been reported. AL and CC are primary biological determinants of refractive error and myopia (Cheng et al., 2013; Chen et al., 2014). In particular, some studies have shown that AL growth can be used as a proxy to predict refractive development at an early age (Tideman et al., 2018; Sanz et al., 2019). Therefore, identifying variants associated with AL and CC in addition to refraction can enhance our understanding of the genetic architecture of refraction (Vergara et al., 2018).

Hence, this study investigated the associations between three novel loci (rs12511037, rs13215566, and rs12206610) and the occurrence and development of myopia, spherical equivalent refraction (SER), AL, and CC in southeastern Chinese schoolchildren during a 4-year follow-up period.

## Materials and Methods

### Study Subjects

The study was a 4-year school-based prospective longitudinal study associated with the Wenzhou Epidemiology of Refractive Error (WERE) project. Among 64 primary schools, we selected three schools using stratified random sampling in the Lucheng district of Wenzhou, southeastern China. The three schools had similar campus cultures, educational qualities and community socioeconomic statuses. Grade 1 children were included in this study. Written informed consent was obtained from each participant. The study was approved by the ethics committee of the Eye Hospital of Wenzhou Medical University and followed the tenets of the Declaration of Helsinki. All participants underwent a complete ophthalmological examination including manifest (non-cycloplegic) refraction (every semester), AL measurement (every year), and CC measurement (every year). The amounts of time spent on near work and outdoors were ascertained from a questionnaire. Near-work activities included doing homework, extracurricular reading and using electronic devices. The amounts of time spent on near work and outdoors per day were calculated as (5 ^∗^ time on weekdays + 2 ^∗^ time on weekends)/7. Myopia was defined as an SER of at least −1.0 diopter (D) (Fan et al., 2011; You et al., 2012; Wu et al., 2015b). Incident myopia was defined as the proportion of children who were non-myopic at baseline but who subsequently developed myopia during the follow-up period. The annual shift in refraction was the difference in mean SER (the follow-up measurement minus the baseline measurement) divided by the mean follow-up time in years. A significant myopic shift was defined as a change in SER ≤ −0.50 D/y (Wu et al., 2015a; Hsu et al., 2017). As the refractive data of both eyes were strongly correlated (Spearman’s ρ = 0.84–0.98, *P* < 0.001), only the data for the right eye were analyzed.

### SNP Selection and Genotyping

A total of three SNPs in three candidate regions were selected for the present study. The selected SNPs are located in the *AREG* (rs12511037), *GABRR1* (rs13215566), and *PDE10A* (rs12206610) genes. The information about these three genes is shown in Table 1. The coordinates and variant identifiers are reported in the NCBI B37 (hg19) genome build and were annotated using the University of California Santa Cruz (UCSC) Genome Browser (Kent et al., 2002). DNA was extracted from oral mucosa for genotyping. Primers were designed for each SNP using Primer 5.0 software. The primer sequences are listed in Table 2.

**TABLE 1:** Information about *PDE10A*, *AREG*, and *GABRR1*.

**SNP**	**Gene**	**Chromosome (hg19)**	**Position**	**Alleles**	**MAF**	**Variant position**
rs12511037	AREG	4	75334864	C > T	0.085	Not found
rs13215566	*GABRR1*	6	89918638	C > G	0.042	Intron Variant
rs12206610	*PDE10A*	6	166016800	C > T	0.068	Intron Variant

**MAF, minor allele frequency; information about the MAF in East Asian cohorts is from the 1000 Genomes Database.**

**TABLE 2:** The primer pairs used for PCR.

**SNP**	**Direction**	**Primer (5′–3′)**
rs12511037	F	TGTAACCCAGTCCCAACTAGAGA
	R	CCAACAGCCCACACATTGTC
rs13215566	F	CGAACACTCCTTGACCCCTG
	R	AACCCGGTGTCTTCAAGTGG
rs12206610	F	GCCATGAAGCTCCTCCATACA
	R	CATGACTGTGGTGAAGTCGC

The SNPs were amplified with PCR using a 2720 Thermal Cycler (Applied Biosystems, Inc. [LongGene], Hangzhou, China). PCRs were performed in 50 μl reaction volumes containing 50 ng of genomic DNA, 2 μl of each 10 μM primer pair, a 0.4 μM final primer concentration and 1 μl of DNA template. Initial denaturation was performed for 2 min at 98°C, followed by 30 cycles of 98°C for 10 s, 56°C for 10 s, and 72°C for 10 s and a final elongation of 2 min at 72°C followed by a hold at 4°C. Genotyping was performed by Sanger DNA sequencing (Applied Biosystems, 3730XL). Sequence alignment was performed using the SeqMan program in DNASTAR software (DNASTAR Inc., Madison, WI, United States).

### Statistical Analysis

Statistical analysis was performed by SNPtest software for Linux^1^ to identify genetic variants significantly associated with the occurrence and development of myopia, SER and ocular parameters. The additive, dominant, recessive, general and heterozygous models were used in the genetic analyses by comparing major-allele homozygotes vs. heterozygotes vs. minor-allele homozygotes; major-allele homozygotes vs. heterozygotes + minor-allele homozygotes; major-allele homozygotes + heterozygotes vs. minor-allele homozygotes; major-allele homozygotes vs. minor-allele homozygotes; and minor-allele homozygotes + major-allele homozygotes vs. heterozygotes, respectively. The inheritance models were adjusted for sex, age, time spent on near work, and time spent outdoors. Normally distributed data were expressed as means ± standard deviations (SDs) and the skewness of the data was expressed as the median (P25 and P75). The odds ratios (ORs) with corresponding 95% confidence intervals (CIs) were presented. Ten thousand permutations were used for each model to correct for multiple testing. For each SNP, we kept the population sizes of the different groups the same, but interfered with the genotypes 10,000 times, and obtained 10,000 chi-square values of the interference samples. Then we defined the value of *P*′, which equaled the distribution of the original *P*-value in the simulated *P*-values calculated from the actual data. A corrected *P*-value < 0.05 was considered significant.

## Results

### Characteristics of the Study Population

Among the 550 children in grade 1, those without a complete ocular examination (*n* = 17), those who had ocular diseases or who wore contact lenses (*n* = 12), and those without genotype data (*n* = 52) were excluded. After applying the exclusion criteria, 469 children were included for further analyses. Among them, 406 children were non-myopic and 63 children were myopic at baseline. Table 3 presents the characteristics of the participants (*n* = 469). The participants included 249 (53.1%) boys and 220 (46.9%) girls. The mean age of all children was 6.33 ± 0.48 years. At baseline, the SER was 0.04 D (−0.29, 0.46) for all children, and the change in SER was −0.88 D (−1.92, −0.21) during the 4-year follow-up. At baseline, the AL was 22.71 ± 0.70 mm for all children, and the increase in AL was 1.21 mm (0.77, 1.61). At baseline, the CC was 7.79 mm (7.60, 7.97) for all children, and the change in CC was 0.034 mm (0.013, 0.056).

**TABLE 3:** Characteristics of the participants.

**Variables**	
Number	469
Age (years)	6.33 ± 0.48
Male sex, *N* (%)	249 (53.1)
Baseline SER (D), median (Q1, Q3)	0.04 (−0.29, 0.46)
Baseline AL (mm), mean ± SD	22.71 ± 0.70
Baseline CC (mm)	7.79 (7.60, 7.97)
ΔSER (D), median (Q1, Q3)	−0.88 (−1.92, 0.21)
ΔAL (mm), median (Q1, Q3)	1.21 (0.77, 1.61)
ΔCC (mm), median (Q1, Q3)	0.034 (0.013, 0.056)
Near-work time (hours/day)	4.47 ± 1.32
Outdoor time (hours/day)	1.86 ± 0.70

**SER, spherical equivalent refraction; D, diopter; AL, axial length; CC, corneal curvature. Δ is the change in SER, AL or CC during the 4-year follow-up.**

### Associations of Genetic Variants With Myopia and SER

For all children (*n* = 469), the SER changed by −0.22 D (−0.48, −0.05) every year. One hundred eight children (23.0%) had a significant rate of myopic shift. The genotypes and allele frequencies of each SNP associated with a significant myopic shift and a non-significant myopic shift are shown in Table 4. However, after adjusting for age, sex, time spent on near work, and time spent outdoors, we did not find a significant difference in genotype or allele frequency between significant myopic shift and non-significant myopic shift.

**TABLE 4:** Distribution of genotypes and alleles of the three loci in the remaining non-myopic group and incident myopic group.

**SNP**					**SNP model**	**OR (95% CI)**	***P***	***P*′**
***AREG***								
rs12511037	CC	CT	TT	C/T		1.127 (0.855–1.486)		
Remaining non-myopic	71 (0.306)	71 (0.306)	90 (0.388)	0.459/0.541	Additive		0.551	0.562
Incident myopic	56 (0.322)	41 (0.236)	77 (0.442)	0.440/0.560	Dominant		0.945	0.945
					Recessive		0.273	0.275
					General		0.379	0.384
					Heterozygous		0.196	0.198
***GABRR1***								
rs13215566	CC	CG	GG	C/G		1.410 (0.349–5.665)		
Remaining non-myopic	228 (0.983)	4 (0.017)	0 (0)	0.991/0.009	Additive		0.696	0.697
Incident myopic	170 (0.977)	4 (0.023)	0 (0)	0.989/0.011	Dominant		0.696	0.697
					Recessive		–	–
					General		–	–
					Heterozygous		0.696	0.697
***PDE10A***								
rs12206610	CC	CT	TT	C/T		0.870 (0.527–1.438)		
Remaining non-myopic	192 (0.828)	40 (0.172)	0 (0)	0.914/0.086	Additive		0.565	0.55
Incident myopic	147 (0.845)	27 (0.155)	0 (0)	0.922/0.078	Dominant		0.565	0.55
					Recessive		–	–
					General		–	–
					Heterozygous		0.565	0.55

**OR, odds ratio; CI, confidence interval; P, P-value; P′, P-value adjusted by 10,000 permutations. Adjusted for sex, age, near-work time, and outdoor time.**

For children who were non-myopic at baseline (*n* = 406), 42.9% (*n* = 174) had incident myopia, and 57.1% (*n* = 232) remained non-myopic. The frequencies of genotypes and alleles for the three loci in the remaining non-myopic group and incident myopic group are displayed in Table 5. There were no significant associations between the SNPs (rs12511037, rs13215566, and rs12206610) and incident myopia after adjusting for age, sex, time spent on near work, and time spent outdoors.

**TABLE 5:** Distribution of genotypes and alleles of the three loci in the control group and the significant myopic shift group.

**SNP**					**SNP model**	**OR (95% CI)**	***P***	***P*′**
***AREG***								
rs12511037	CC	CT	TT	C/T		1.037 (0.763–1.408)		
△SER > −0.50 D/y	112 (0.310)	100 (0.277)	149 (0.413)	0.449/0.551	Dominant		0.681	0.679
△SER ≤ −0.50 D/y	35 (0.324)	25 (0.231)	48 (0.445)	0.440/0.560	Recessive		0.656	0.661
					Heterozygous		0.347	0.35
					Additive		0.971	0.972
					General		0.642	0.647
***GABRR1***								
rs13215566	CC	CG	GG	C/G		0.740 (0.159–3.453)		
△SER > −0.50 D/y	353 (0.978)	7 (0.019)	1 (0.003)	0.988/0.012	Dominant		0.758	0.754
△SER ≤ −0.50 D/y	106 (0.981)	2 (0.019)	0 (0)	0.990/0.009	Recessive		–	–
					Heterozygous		0.851	0.722
					Additive		0.692	0.643
					General		0.824	0.976
***PDE10A***								
rs12206610	CC	CT	TT	C/T		1.022 (0.589–1.773)		
△SER > −0.50 D/y	302 (0.837)	59 (0.163)	0 (0)	0.918/0.082	Dominant		0.982	0.99
△SER ≤ −0.50 D/y	90 (0.833)	18 (0.167)	0 (0)	0.917/0.083	Recessive		–	–
					Heterozygous		0.982	0.99
					Additive		0.982	0.99
					General		−	−

**SER, spherical equivalent refraction; ΔSER, annual shift in refraction; OR, odds ratio; CI, confidence interval; P, P-value; P′, p value adjusted by 10,000 permutations. A significant myopic shift was defined as ΔSER* ≤ −*0.50 D/y. Adjusted for sex, age, near-work time, and outdoor time.**

**FIGURE 1:**
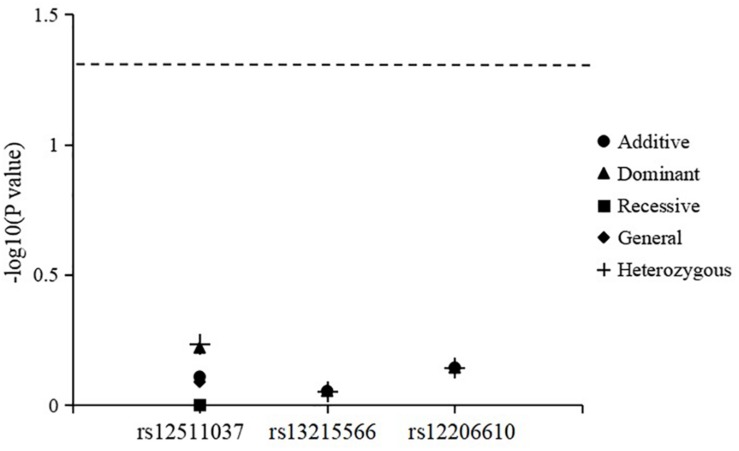
Association of genotypes and alleles of the three loci with the change in spherical equivalent refraction.

The results for genotype and allele associations of the three SNPs with the change in SER are summarized in Figure 1. After adjusting for age, sex, time spent on near work, and time spent outdoors, neither the genotypes nor allele frequencies of the three SNPs were associated with the change in SER.

### Associations of Genetic Variants With Ocular Parameters

**FIGURE 2:**
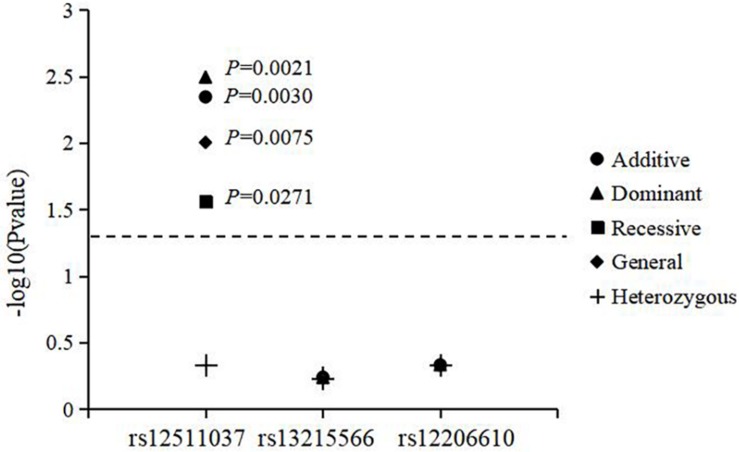
Association of genotypes and alleles of the three loci with the increase in axial length.

The associations of genotypes and alleles of the three loci with the increase in AL are shown in Figure 2 and Table 6. For *AREG* (rs12511037), the *P*-values for the dominant, recessive, additive, and general models were 0.0021, 0.0271, 0.0030, and 0.0075, respectively, after adjusting for age, sex, near-work time, and outdoor time. After 10,000 permutations, the genotype of rs12511037 was still associated with the increase in AL (*P*′ = 0.0032, *P*′ = 0.0275, *P*′ = 0.0045, and *P*′ = 0.0099, respectively). This showed that the T allele and TT genotype were significantly associated with an increase in AL. Children with the TT genotype of rs12511037 had significantly greater ALs (1.19 mm) than those carrying the CC (1.10 mm) or CT (1.08 mm) genotype.

**TABLE 6:** Distribution of the change of axial length and corneal curvature in different genotypes of the three loci.

**SNP**	***n* (%)**	**ΔAL (mm)**	**SNP model**	***P***	***P*′**	**ΔCC (mm)**	**SNP model**	***P***	***P*′**
***AREG***									
rs12511037									
CC	147 (31.3)	1.10 (0.75, 1.56)	Dominant	0.0021	0.0032	0.035 (0.014, 0.056)	Dominant	0.744	0.749
CT	125 (26.7)	1.08 (0.70, 1.49)	Recessive	0.0271	0.0275	0.033 (0.013, 0.052)	Recessive	0.302	0.306
TT	197 (42.0)	1.19 (0.84, 1.64)	Heterozygous	0.46	0.47	0.034 (0.014, 0.059)	Heterozygous	0.421	0.426
C/T	0.447/0.553		Additive	0.0030	0.0045		Additive	0.437	0.559
			General	0.0075	0.0099		General	0.558	0.564
***GABRR1***									
rs13215566									
CC	459 (97.9)	1.10 (0.78, 1.61)	Dominant	0.575	0.578	0.037 (0.013, 0.056)	Dominant	0.583	0.390
CG	9 (1.9)	1.15 (0.85, 2.05)	Recessive	–	–	0.053 (0.025, 0.071)	Recessive	–	–
GG	1 (0.2)	1.18 (1.18, 1.18)	Heterozygous	0.575	0.581	0.06 (0.06, 0.06)	Heterozygous	0.583	0.390
C/G	0.989/0.012		Additive	0.575	0.575		Additive	0.583	0.390
			General	–	–		General	–	–
***PDE10A***									
rs12206610									
CC	392 (83.6)	1.1 (0.78, 1.61)	Dominant	0.456	0.464	0.033 (0.013, 0.056)	Dominant	0.0070	0.0096
CT	77 (16.4)	1.12 (0.78, 1.63)	Recessive	–	–	0.059 (0.025, 0.071)	Recessive	–	–
TT	0 (0)	–	Heterozygous	0.456	0.464	–	Heterozygous	0.0070	0.0096
C/T	0.918/0.082		Additive	0.456	0.464		Additive	0.0070	0.0096
			General	–	–		General	–	–

**AL, axial length; CC, corneal curvature; Δ is the change in AL or CC during the 4-year follow-up; P, P-value; P′, p-value adjusted by 10,000 permutations. Adjusted for sex, age, near-work time, and outdoor time.**

**FIGURE 3:**
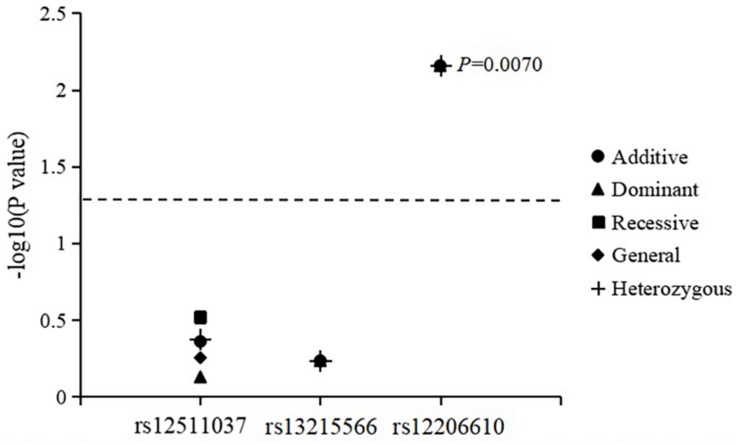
Association of genotypes and alleles of the three loci with the increase in corneal curvature.

The associations of genotypes and alleles of the three SNPs with the change in CC are summarized in Figure 3 and Table 6. After adjusting for age, sex, near-work time, and outdoor time, the genotype of *PDE10A* (rs12206610) was associated with the change in CC in the additive (*P* = 0.0070), dominant (*P* = 0.0070), and heterozygous models (*P* = 0.0070). After 10,000 permutations, the genotype of rs12206610 was still associated with the change in CC in these three models (all *P*′-values = 0.0096).

## Discussion

We performed a genetic association analysis of the occurrence and development of myopia, the change in SER, the increase in AL, and the change in CC in 469 grade 1 schoolchildren during a 4-year follow-up as part of the WERE project. We confirmed that *AREG* (rs12511037) was associated with the increase in AL and that *PDE10A* (rs12206610) was associated with the change in CC.

Several studies have reported genes associated with AL (Fan et al., 2012; Cheng et al., 2013; Miyake et al., 2015). Miyake et al. (2015) identified *WNT7B* as a novel susceptibility gene for AL in Chinese individuals. Cheng et al. (2013) found nine significant loci for AL (*RSPO1*, *C3orf26*, *LAMA2*, *GJD2*, *ZNRF3*, *CD55*, *MIP*, *ALPPL2*, and *ZC3H11B*) in a study of 12,531 Europeans and 8,216 Asians. Fan et al. (2016) reported that *AREG* (rs12511037) was associated with myopia in Asian populations. No prior studies reported an association between *AREG* and AL. Therefore, we performed a 4-year cohort study and found that *AREG* was significantly associated with the increase in AL. Song et al. (2018) reported that the injection of the *AREG* antibody into guinea pigs resulted in a statistically significant decrease in AL, which confirms our finding in animal experiments. The *AREG* gene is a ligand of the epidermal growth factor receptor (EGFR), which promotes the growth of normal epithelial cells (Wang et al., 2004). It is specifically expressed in human retinal pigment epithelium (RPE). The relationship between the RPE and myopia is particularly close. During the formation of myopia, RPE cells not only undergo significant structural changes but also transmit signals of myopia in the retina, regulate the growth of sclera cells, and thus regulate the growth and development of the eyeball (Wallman, 1990). We speculated that *AREG* may bind to EGFR and use RPE cells as a hub to activate specific signaling pathways to induce myopia, mainly manifested in the growth of AL among the biological parameters. However, the function of the *AREG* gene in myopia remains to be further studied.

Although *AREG* (rs12511037) demonstrated evidence of an association with AL, it was not associated with SER in our study. In eyes without refractive error, AL and CC are precisely scaled relative to one another and have a strong phenotypic correlation (Guggenheim et al., 2013). Therefore, *AREG* might mediate compensatory effects through changes in CC or optical power, thereby balancing their impacts on SER. In addition, AL measurements are more precise and less prone to errors than cycloplegic or non-cycloplegic assessments of refraction (Fan et al., 2012).

Some genes associated with CC have been detected (Han et al., 2011; Chen et al., 2014; Miyake et al., 2015; Vergara et al., 2018). Vergara et al. (2018) confirmed associations of two previously known loci (rs2114039 and rs634990) with CC in 1,871 European-Americans. Miyake et al. (2015) identified *WNT7B* (rs10453441) as a novel susceptibility gene for CC in Chinese individuals. We found that *PDE10A* (rs12206610) was associated with the change in CC, not with the change in AL. This finding may indicate that *PDE10A* acts on the irregularities of the cornea rather than on the length of the eyeball. However, correlation does not imply causation. *PDE10A* is mostly expressed in the retina (Wagner et al., 2013) and is involved in circadian rhythm, and the levels of the *PDE10A* protein display circadian rhythm at retinal photoreceptors (Wolloscheck et al., 2011), suggesting potential roles of this protein in the visual circle. *PDE10A* has gained attention as a therapeutic target for psychiatric disorders (Wilson and Brandon, 2015; Zhang et al., 2017; Cheng et al., 2018). Few studies have reported an association between *PDE10A* and myopia, and no prior study has analyzed the relationship between *PDE10A* and ocular parameters. Considering the small samples in this study, the relationship between *PDE10A* and CC needs to be further investigated and the underlying biological mechanism needs to be clarified in both human genetic epidemiology studies and animal models.

One strength of this study is that it is the first to analyze associations between these three SNPs and refraction and ocular parameters during a cohort study of Chinese schoolchildren. Nevertheless, this study has some limitations that should be acknowledged. First, the population in our study was limited to the southeastern Chinese population. As a result, generalization of the results is limited to some extent. Second, the sample size of our study was small. Hence, further cohort studies with a larger sample size are warranted.

## Conclusion

We identified *AREG* (rs12511037) and *PDE10A* (rs12206610) as new susceptibility loci for the increase in AL and change in CC, respectively, in Chinese schoolchildren. This finding provides a basis for further exploration of *AREG* and *PDE10A* involvement in the development of myopia and its molecular mechanism. The role of *AREG* and *PDE10A* in the pathogenesis of myopia requires further studies in animal models and human genetic epidemiology.

## Data Availability Statement

The datasets analyzed in this study are available from the corresponding author (YC, cyy@mail.eye.ac.cn) upon reasonable request.

## Ethics Statement

The studies involving human participants were reviewed and approved by the Ethics Committee of the Eye Hospital of Wenzhou Medical University. The participants provided written informed consent to participate in this study.

## Author Contributions

YL wrote the manuscript. YL and YD performed the data analyses. DJ, CL, and YC helped to perform the analyses with constructive discussions. XH, LL, and HX helped to revise the manuscript. BV helped to polish the English language and grammar. YC contributed to the conception of the study.

## Conflict of Interest

The authors declare that the research was conducted in the absence of any commercial or financial relationships that could be construed as a potential conflict of interest.

## Footnotes

1 http://mathgen.stats.ox.ac.uk/genetics_software/snptest/snptest.html
